# Habitual Levels of High, But Not Moderate or Low, Impact Activity Are Positively Related to Hip BMD and Geometry: Results From a Population-Based Study of Adolescents

**DOI:** 10.1002/jbmr.1631

**Published:** 2012-04-10

**Authors:** Kevin Deere, Adrian Sayers, Jörn Rittweger, Jon H Tobias

**Affiliations:** 1 Musculoskeletal Research Unit, School of Clinical Sciences, University of BristolBristol, UK; 2 Division of Space Physiology, Institute of Aerospace Medicine, German Aerospace CenterCologne, Germany; 3 Institute for Biomedical Research into Human Movement and Health, Manchester Metropolitan UniversityManchester, UK

**Keywords:** ACCELEROMETER, BONE MINERAL DENSITY, IMPACT LOADING, ALSPAC

## Abstract

Whether a certain level of impact needs to be exceeded for physical activity (PA) to benefit bone accrual is currently unclear. To examine this question, we performed a cross-sectional analysis between PA and hip BMD in 724 adolescents (292 boys, mean 17.7 years) from the Avon Longitudinal Study of Parents and Children (ALSPAC), partitioning outputs from a Newtest accelerometer into six different impact bands. Counts within 2.1 to 3.1*g*, 3.1 to 4.2*g*, 4.2 to 5.1*g*, and >5.1*g* bands were positively related to femoral neck (FN) BMD, in boys and girls combined, in our minimally adjusted model including age, height, and sex (0.5–1.1*g*: beta = −0.007, *p* = 0.8; 1.1–2.1*g*: beta = 0.003, *p* = 0.9; 2.1–3.1*g*: beta = 0.042, *p* = 0.08; 3.1–4.2*g*: beta = 0.058, *p* = 0.009; 4.2–5.1*g*: beta = 0.070, *p* = 0.001; >5.1*g*: beta = 0.080, *p* < 0.001) (beta = SD change per doubling in activity). Similar positive relationships were observed between high-impact bands and BMD at other hip sites (ward's triangle, total hip), hip structure indices derived by hip structural analysis of dual-energy X-ray absorptiometry (DXA) scans (FN width, cross-sectional area, cortical thickness), and predicted strength (cross-sectional moment of inertia). In analyses where adjacent bands were combined and then adjusted for other impacts, high impacts (>4.2*g*) were positively related to FN BMD, whereas, if anything, moderate (2.1–4.2*g*) and low impacts (0.5–2.1*g*) were inversely related (low: beta = −0.052, *p* = 0.2; medium: beta = −0.058, *p* = 0.2; high: beta = 0.137, *p* < 0.001). Though slightly attenuated, the positive association between PA and FN BMD, confined to high impacts, was still observed after adjustment for fat mass, lean mass, and socioeconomic position (high: beta = 0.096, *p* = 0.016). These results suggest that PA associated with impacts >4.2*g*, such as jumping and running (which further studies suggested requires speeds >10 km/h) is positively related to hip BMD and structure in adolescents, whereas moderate impact activity (eg, jogging) is of little benefit. Hence, PA may only strengthen lower limb bones in adolescents, and possibly adults, if this comprises high-impact activity. © 2012 American Society for Bone and Mineral Research.

## Introduction

Mechanical strain is an important determinant of skeletal growth and modeling. For example, animal studies have demonstrated that bone strain (ie, deformation relative to bone length) stimulates bone formation in proportion to its rate and magnitude.^1^, ^2^ Bone strain is directly related to the strength of applied force, which for the lower limbs comprises ground reaction forces generated by the musculature during locomotion, with body mass serving as resistance.^3^, ^4^ There has been considerable interest in the effect of physical activity (PA) on bone development in childhood, and in particular whether increased weight-bearing activities during this time results in a higher peak bone mass and strength of the lower limb, thereby reducing the risk of osteoporotic hip fracture in later life. For example, in 22 trials of effects of weight-bearing exercise interventions on bone mineral accrual in childhood, generally positive effects were observed, particularly during early puberty.^5^

In terms of cross-sectional studies examining relationships with habitual levels of PA, in a pQCT-based study of 1068 eighteen-year-old men by Lorentzon and colleagues,^6^ a minimum threshold of 4 hours per week participation in sporting activity was identified for influencing cortical bone size. However, questionnaires in which participants provide information about day-to-day physical activity and exercise may be unreliable, particularly when applied to child populations,^7^ in which case accelerometry may provide a more objective measure of physical activity. For example, an Actigraph device has been employed in the Avon Longitudinal Study of Parents and Children (ALSPAC), physical activity being defined as light, moderate, or vigorous, based on thresholds of 3600 and 6200 counts per minute, reflecting the transition from normal to brisk walking, and brisk walking to jogging, respectively.^8^ Based on this approach, we found that vigorous day-to-day PA is associated with increased cortical bone mass in adolescents, through a combination of increased periosteal growth and reduced endocortical resorption, whereas light or moderate PA has no detectable association.^9^ These findings suggest that a threshold of strain needs to be exceeded to affect bone development. However, the upper range of acceleration that can be detected by the Actigraph is 2.5*g*,^10^ implying this device is unable to distinguish higher-impact activities such as jumping, in which accelerations generally exceed 5*g*.^11^

To provide more information about the relationship between level of impact associated with physical activity and bone outcomes, a Newtest accelerometer (Newtest Oy, Oulu, Finland) was developed to measure impacts across a wide range of *g*-bands. Using this approach, in 64 premenopausal women enrolled in an exercise intervention study, gain in femoral neck (FN) bone mineral density (BMD) was positively related to PA associated with impacts of >3.9*g*, whereas no relationship was seen for lesser impacts.^11^ This raises the possibility that activities such as jogging, associated with impacts between 2.5 and 3.8*g*, may not influence bone development, and that exposure to higher strains is needed such as those associated with running and jumping. In a subsequent work based on analysis of contemporaneous pQCT scans, high-impact PA was related to an increase in bone circumference and cortical thickness, suggesting a combination of enhanced periosteal growth and reduced endocortical resorption.^12^

We investigated the role of exposure to impacts related to PA in skeletal development, by performing a cross-sectional study in a population-based sample of adolescents from ALSPAC, who were asked to wear a Newtest device for up to 7 days while performing normal day-to-day activities. In this work, we report associations between number of impacts within different *g* bands, hip BMD, and hip geometry as assessed by hip structural analysis (HSA). In particular, we wished to determine 1 whether hip BMD is positively related to the number of impacts associated with PA; 2 whether this association persists after adjusting for a range of potential confounding factors including fat mass, lean mass, and socioeconomic position; 3 whether, according to the data we find, that the associations between impacts and hip BMD result from relationships with overall bone size and/or cortical thickness; and 4 whether a threshold of impact needs to be exceeded before a positive relationship with hip indices is observed.

## Subjects and Methods

### Study participants

ALSPAC is a geographically based birth cohort study investigating factors influencing the health, growth, and development of children. All pregnant women resident within a defined part of the former county of Avon in South West England with an expected date of delivery between April 1991 and December 1992 were eligible for recruitment, of whom 14,541 were enrolled^13^ (http://www.bristol.ac.uk/alspac). Ethical approval was obtained from the ALSPAC Law and Ethics committee and relevant local ethics committees, written informed consent was provided by all parents, and young people provided written assent. Data in ALSPAC is collected by self-completion postal questionnaires sent to parents, by linkage to computerized records, by abstraction from medical records, and from examination of the children at research clinics. The present study was based on the research clinic held at approximately 17 years of age, between December 2008 and June 2011, to whom all ALSPAC participants were invited.

### PA measurements

Those who agreed to participate in the accelerometer substudy, subject to availability, were fitted with a version of the Newtest monitor produced for this study, which recorded accelerations within 33 separate bands across the range 0.3 to 9.9*g* above gravitational force (1*g*), as previously described for other research applications.^11^ Participants were asked to wear it for 7 consecutive days during waking hours, recharge it overnight, and only take it off at other times off for contact sports or when it might get wet. Participants were also asked to record a diary when the monitor was worn, a valid recording being defined as a minimum of 8 hours recording per day for 2 days. Raw data was read into Stata 11 using custom-designed code. Number of counts per subject was subsequently calculated, expressed as number of counts/day across six impact bands namely, 0.5 to 1.1*g*, 1.1 to 2.1*g*, 2.1 to 3.1*g*, 3.1 to 4.2*g*, 4.2 to 5.1*g*, and >5.1*g*. These related to normal walking (0.5–1.1*g*), brisk walking (1.1–2.1*g*), jogging/running (2.1–5.1*g*), and jumping (>5.1*g*), as determined by our previous calibration study based on a separate group of 22 school children (mean 17.1 years, 15 boys) who were asked to wear Newtest monitors while performing a series of supervised activities (K Deere and colleagues, unpublished results).

### Measurement of hip structure

All children attending the research clinic at age 17 years were offered a left hip dual-energy X-ray absorptiometry (DXA) scan on a GE Lunar Prodigy, generating total hip (TH) and FN BMD (g/cm^2^). Each scan was analyzed using the manufacturer's automated advanced hip analysis (AHA) software, which generated a range of structural parameters at the site of minimum FN width, as described.^14^ Geometric indices consisted of femoral neck width (FNW, mm), cortical cross-sectional area (CSA, cm^2^), and cortical thickness (CT, mm). Derived biomechanical strength indices comprised buckling ratio (BR) (0.5*FNW divided by CT), cross-sectional moment of inertia (CSMI, cm^4^), which reflects resistance to bending, and section modulus (SM, cm^3^), which is CSMI adjusted for size by dividing by FN radius.

### Confounders

Data on lean mass and fat mass were obtained from total body DXA scans performed on a Lunar Prodigy at the same clinic visit. Height was measured using a Harpenden stadiometer (Holtain Ltd., Crymych, UK). Maternal social class was derived from self-report questionnaire administered at 32 weeks gestation.

### Statistical methods

Descriptive data was expressed as medians with 25th and 75th interquartile range (IQR). Regression analysis was used to examine relationships between number of counts per day within each band, and hip parameters. We used a nonparametric bootstrap sampled with replacement based on 1,000 replicates to generate beta coefficients with 95% confidence intervals. Activity data was first normalized by log transformation. Eight different models were used: (1) minimal, adjusted for age, height, and sex; (2) 1 + fat and lean mass; (3) 2 + socioeconomic position; (4) 1 + other activity bands; (5) 4 + fat mass; (6) 4 + lean mass; (7) 4 + fat and lean mass; and (8) 7 + socioeconomic position. All analyses were performed by KD, in Stata 11.2 (College Station, TX, USA).

## Results

### Participant characteristics

Of the 5084 adolescents attending the age 17 years ALSPAC research clinic, 3925 were asked if they would like to wear an accelerometer, of whom 2472 agreed, which was available for 1390 participants. Of the 1175 subjects who returned the monitor, the monitor was damaged or nonfunctional in 22 cases, returned unworn in 189 cases, and returned either without a completed diary or with a diary indicating this has been worn for less than 8 hours per day for 2 days (*n* = 232). This left 756 participants with valid recordings, of whom 724 ALSPAC participants (295 boys) also had information about hip BMD and other covariates, who formed the basis of the present analysis. Those included in this study had a mean age of 17.7 years.

As expected, boys were taller and had greater lean mass, whereas fat mass was higher in girls (Table 1). TH BMD and FN BMD were greater in boys, as was FNW, CT, and CSMI. Participants had similar indices of body composition compared to the remainder of the cohort, but higher socioeconomic status (as assessed by maternal social class) as exemplified by a greater proportion in class I and a lower proportion in class V (Supplementary Table 1). Accelerometers were worn for a mean of 5.8 days. The median number of counts accrued per day in the six different activity bands is shown in Table 2. There was a profound fall in the number of counts upon moving from lower- to higher-impact activity. The number of counts appeared to be greater in boys compared to girls, particularly for higher-impact activity.

**Table 1:** Characteristics of Participants, Including Hip Parameters

Variable	Sex	Mean	(SD)	Median	p25	p75
Age (years)	M	17.7	(0.27)	17.6	17.7	17.8
	F	17.7	(0.30)	17.6	17.7	17.8
Height (cm)	M	178.7	(6.93)	174.2	178.0	183.2
	F	164.9	(5.76)	161.2	164.5	168.9
Fat mass (kg)	M	13.4	(9.49)	6.9	10.2	17.1
	F	21.7	(9.15)	15.1	20.0	25.8
Lean mass (kg)	M	54.8	(5.86)	50.5	54.6	58.5
	F	38.1	(3.97)	35.3	37.7	40.6
Total hip BMD (g/cm^2^)	M	1.17	(0.14)	1.08	1.16	1.3
	F	1.06	(0.13)	0.96	1.05	1.1
Femoral neck BMD (g/cm^2^)	M	1.13	(0.16)	1.04	1.11	1.2
	F	1.05	(0.12)	0.96	1.04	1.1
Minimum femoral neck width (mm)	M	32.96	(3.29)	31.23	32.88	34.8
	F	27.98	(2.48)	26.67	28.09	29.4
Cortical thickness (mm)	M	1.98	(0.27)	1.82	1.95	2.1
	F	1.90	(0.24)	1.73	1.89	2.1
Cross-sectional moment of inertia (cm^4^)	M	2.60	(0.74)	2.07	2.49	3.0
	F	1.52	(0.38)	1.24	1.48	1.8

Values are mean (SD), median, lower and upper quartiles (*n* = 724; males = 292, females = 432).

M = male; F = female; p25 = 25th percentile; 75th percentile; BMD = bone mineral density.

**Table 2:** Number of Impacts According to *g*-Band

*g*-Band	Sex	Median per day	p25	p75
0.5 up to 1.1*g*	M	3600.0	2028.2	5749.7
	F	3121.0	1978.6	4778.8
1.1 up to 2.1*g*	M	755.0	384.5	1259.7
	F	596.6	318.4	1083.7
2.1 up to 3.1*g*	M	107.0	53.1	201.5
	F	83.6	40.5	170.1
3.1 up to 4.2*g*	M	33.2	16.5	68.0
	F	25.0	11.5	51.1
4.2 up to 5.1*g*	M	10.1	5.0	24.5
	F	7.8	3.1	15.5
>5.1*g*	M	13.3	6.7	28.6
	F	8.6	4.2	18.6

Data for boys (*n* = 292) and girls (*n* = 432), expressed as median number of counts (with 25th/75th percentile) per day.

p25 = 25th percentile; p75 = 75th percentile; M = male; F = female.

### Activity versus hip parameters: minimally adjusted analyses

**Fig. 1:**
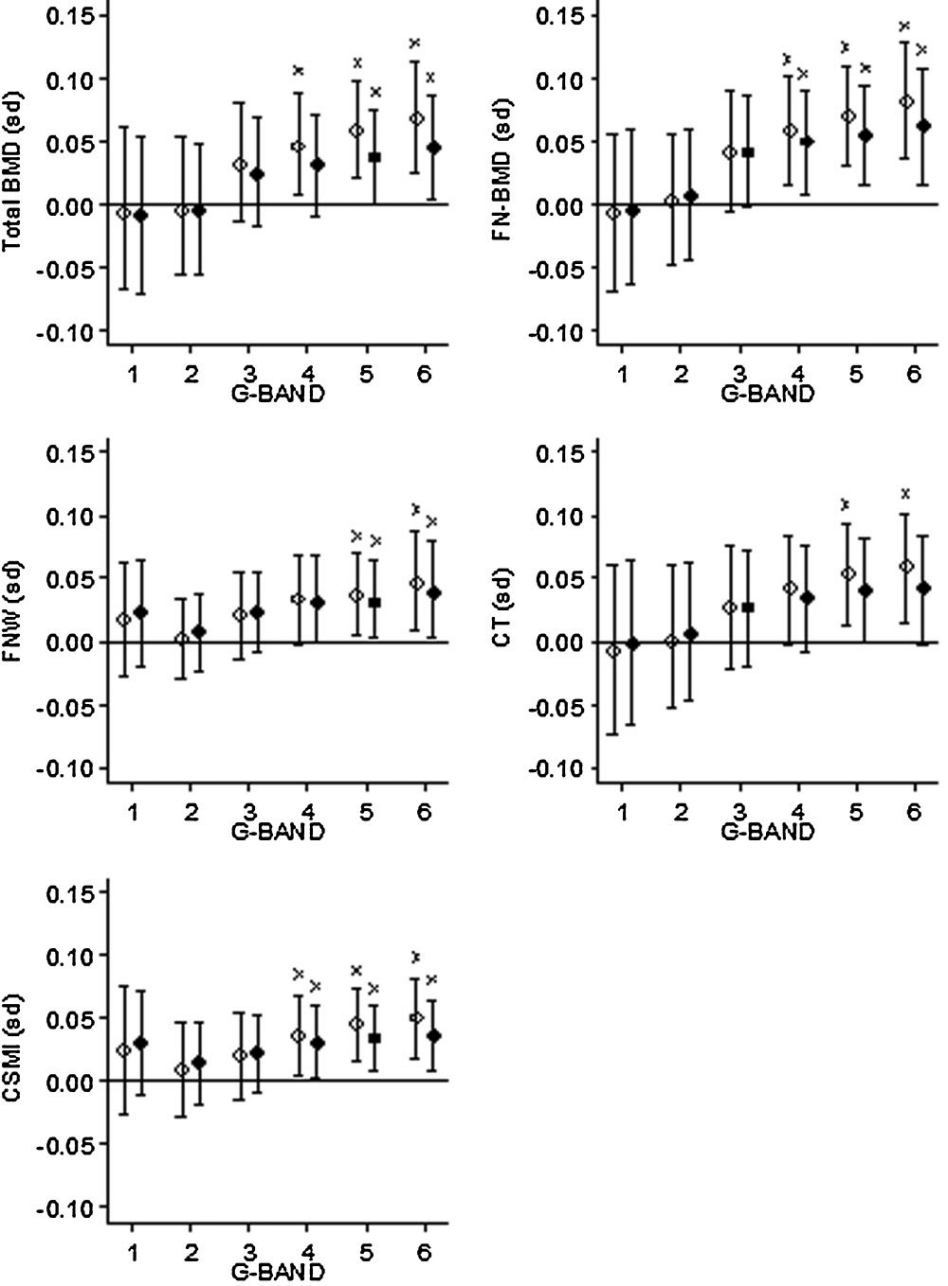
Results of regression between number of impacts within different activity bands, and hip parameters, in 724 boys and girls combined. Data are shown as beta coefficients (change in SD per doubling in activity) with 95% confidence intervals, based on six different *g* bands (1 = 0.5–1.1g; 2 = 1.1–2.1*g*; 3 = 2.1–3.1*g*; 4 = 3.1–4.2*g*; 5 = 4.2–5.1*g*; 6 = > 5.1*g*) (bootstrap estimates derived from 1000 replicates). Total = total hip BMD; FN = femoral neck; FNW = femoral neck width; CT = cortical thickness; CSMI = cross sectional moment of inertia. *x* = *p* < 0.05. (○) Basic model (adjusted for age, height, and gender). (•) Additionally adjusted for fat and lean mass.

Counts within 3.1 to 4.1*g*, 4.1 to 5.1*g*, and >5.1*g* bands were positively related to FN BMD, in boys and girls combined, in our minimally adjusted model including age, height, and sex (see Model 1, Table 3). On comparing beta coefficients across all six bands, there was evidence of a dose-response relationship, with a progressive rise in coefficients starting at band 3, namely 2.1 to 3.1*g* (Fig. 1). Equivalent relationships were observed between activity and TH BMD.

**Table 3:** Results of Regression Between Number of Impacts Within Six Distinct *g*-Bands and FN BMD

		Model 1 = (age, height, gender)				Model 2 = (Model 1 + fat and lean mass)				Model 3 = (Model 2 + social position)			
				
*g*-Band	*g* Range	*R*^2^	beta	95%CI	*p*	*R*^2^	beta	95%CI	*p*	*R*^2^	beta	95%CI	*p*
1	0.5–1.1*g*	0.110	–0.007	(−0.069, 0.056)	0.821	0.244	–0.004	(−0.064, 0.058)	0.889	0.246	–0.009	(−0.075, 0.057)	0.781
2	1.1–2.1*g*	0.109	0.003	(−0.048, 0.056)	0.911	0.244	0.007	(−0.044, 0.060)	0.787	0.246	0.000	(−0.055, 0.054)	0.996
3	2.1–3.1*g*	0.113	0.042	(−0.005, 0.090)	0.081	0.247	0.041	(−0.003, 0.086)	0.076	0.248	0.031	(−0.018, 0.080)	0.208
4	3.1–4.2*g*	0.118	0.058	(0.015, 0.101)	0.009	0.250	0.049	(0.008, 0.090)	0.023	0.251	0.043	(−0.006, 0.091)	0.073
5	4.2–5.1*g*	0.123	0.070	(0.031, 0.109)	0.001	0.252	0.055	(0.016, 0.095)	0.007	0.253	0.047	(0.001, 0.092)	0.039
6	>5.1*g*	0.127	0.080	(0.035, 0.127)	<0.001	0.254	0.062	(0.016, 0.107)	0.006	0.257	0.060	(0.010, 0.112)	0.017

Data from 724 boys and girls combined (bootstrap estimates derived from 1000 replicates). Analysis shows beta coefficient (SD change in FN BMD per doubling in activity), 95% CI, *p* values. and *R*^2^. Model 1 is adjusted for age, height, and gender; model 2 as for model 1 additionally adjusted for fat and lean mass; model 3 as for model 2 additionally adjusted for social position (*n* = 588). Tests for gender interaction all *p* > 0.05.

FN = femoral neck; BMD = bone mineral density; CI = confidence interval.

In terms of relationships between activity and hip structure, positive associations were observed between the number of counts within the two top bands, namely 4.1 to 5.1*g* and >5.1*g*, and FNW and CT (Fig. 1). On comparing beta coefficients between all bands, a similar dose-response pattern was observed to that seen for BMD. The relationship between activity and CSMI was similar to the relationship observed for FNW and for CSA (data not shown), with the exception that there was slightly stronger evidence for a positive association with band 4. Equivalent associations were observed for SM (data not shown). There was weak evidence of reduced BR with increasing activity, particularly for higher *g*-bands, but these associations were all *p* > 0.05 (data not shown). Though beta coefficients for hip BMD and structural parameters were slightly higher for girls compared to boys, formal gender interaction tests were all *p* > 0.05.

### Activity versus FN BMD: further models

#### Adjustment for body composition and socioeconomic position

The positive association between PA and FN BMD in our minimally adjusted model was slightly attenuated after adjustment for fat and lean mass, as evidenced by a reduction in beta coefficients of approximately 25%, for associations between FN BMD and *g* bands 4, 5, and 6 (Model 2, Table 3). After additional adjustment for socioeconomic position, little further change was observed in beta coefficients, though there was some diminution in the strength of associations as judged by *p* values, reflecting the reduced study sample (Model 3). An equivalent effect of adjustment for fat and lean mass was observed for TH BMD, and for strength indices as illustrated by FNW, CT, and CSMI (Fig. 1). Although beta coefficients were mildly attenuated after adjusting for fat and lean mass, there was still reasonable evidence for a persisting association; with the exception of CT, for which confidence limits now overlapped with zero.

#### Adjustment for other activity bands: two-band models

Although our results suggest there is a threshold whereby FN BMD is only related to impacts beyond 2.1*g*, impacts above 2.1*g* may still be subthreshold, and only show a positive relationship with BMD due to correlation with higher-impact bands. To provide a more accurate estimate of any threshold value, we compared associations between FN BMD and impacts above or below putative thresholds of 2.1*g*, 3.1*g*, 4.2*g*, and 5.1*g*, adjusted for other impacts (Model 4, Table 4). Irrespective of the precise threshold, impacts below the threshold were inversely related to FN BMD, in contrast to impacts above the threshold, which were positively related. Increasing the threshold from 2.1*g* to 3.1*g* to 4.2*g* resulted in a progressive improvement in model fit as judged by *R*^2^ (0.122, 0.128, and 0.133, respectively), and increase in beta coefficients for high impacts versus FN BMD (0.090, 0.099, and 0.104, respectively), whereas there was little trend in beta coefficient for low impacts (−0.083, −0.086, and −0.083, respectively). Increasing the threshold to 5.1*g* led to no further improvement in model fit, but a decrease in both beta coefficients.

**Table 4:** Relationships Between Femoral Neck BMD and the Number of Counts Within Low/High *g*-Bands Based on Different *g* Cut-points

		Low			High			
			
Model	*g* Cut-point	beta	95%CI	*p*	beta	95%CI	*p*	*R*^2^
1	2.1	−0.005	−0.0668, 0.0595	0.888	0.055	0.0092, 0.1006	0.021	0.116
	3.1	−0.003	−0.0659, 0.0621	0.927	0.070	0.0245, 0.1142	0.002	0.122
	4.2	−0.002	−0.0649, 0.0636	0.955	0.079	0.0339, 0.1218	<0.001	0.126
	5.1	−0.001	−0.0647, 0.0645	0.972	0.080	0.0353, 0.1272	<0.001	0.127
4	2.1	−0.083	−0.1674, 0.0035	0.048	0.090	0.0254, 0.1490	0.003	0.122
	3.1	−0.086	−0.1621, −0.0058	0.029	0.099	0.0423, 0.1524	<0.001	0.128
	4.2	−0.083	−0.1514, −0.0073	0.028	0.104	0.0493, 0.1517	<0.001	0.133
	5.1	−0.076	−0.1436, −0.0032	0.040	0.102	0.0469, 0.1526	<0.001	0.133
5	2.1	−0.066	−0.1507, 0.0190	0.117	0.095	0.0318, 0.1539	0.001	0.151
	3.1	−0.064	−0.1413, 0.0164	0.108	0.099	0.0423, 0.1513	<0.001	0.157
	4.2	−0.058	−0.1329, 0.0194	0.126	0.100	0.0457, 0.1480	<0.001	0.159
	5.1	−0.051	−0.1232, 0.0230	0.175	0.097	0.0411, 0.1471	<0.001	0.159
6	2.1	−0.075	−0.1498, 0.0021	0.054	0.073	0.0153, 0.1297	0.012	0.245
	3.1	−0.074	−0.1419, 0.0018	0.046	0.077	0.0234, 0.1300	0.004	0.249
	4.2	−0.070	−0.1361, 0.0033	0.048	0.079	0.0242, 0.1262	0.002	0.251
	5.1	−0.065	−0.1312, 0.0084	0.061	0.078	0.0235, 0.1278	0.002	0.251
7	2.1	−0.067	−0.1413, 0.0115	0.087	0.077	0.0201, 0.1333	0.008	0.253
	3.1	−0.064	−0.1373, 0.0143	0.088	0.079	0.0237, 0.1308	0.003	0.256
	4.2	−0.059	−0.1268, 0.0173	0.102	0.079	0.0245, 0.1258	0.002	0.257
	5.1	−0.034	−0.0953, 0.0313	0.310	0.087	0.0416, 0.1311	<0.001	0.229
8	2.1	−0.066	−0.1485, 0.0160	0.112	0.067	0.0059, 0.1279	0.032	0.254
	3.1	−0.069	−0.1481, 0.0083	0.083	0.073	0.0164, 0.1305	0.012	0.258
	4.2	−0.065	−0.1364, 0.0082	0.082	0.075	0.0202, 0.1301	0.007	0.260
	5.1	−0.049	−0.1178, 0.0184	0.153	0.057	0.0129, 0.0996	0.011	0.335

Data are for 724 boys and girls combined. Data is shown as beta (SD change in BMD per doubling of activity) with 95%CI, *p* values and *R*^2^ (for upper band in the case of model 1, and for overall fit for models 4, 5, and 6) (bootstrap estimates derived from 1000 replicates). Model 1 is adjusted for age, height, and gender; model 4 is model 1 + physical activity; model 5 is model 4 + fat mass; model 6 is model 4 + lean mass; model 7 is model 4 + fat and lean mass; model 8 is model 7 + social position (*n* = 588). Tests for gender interaction all *p* > 0.05.

BMD = bone mineral density; CI = confidence interval.

The positive association between FN BMD and high-impact activity was unaffected by further adjustment for fat mass (Model 5), but attenuated by approximately 25% after adjustment for lean mass either alone (Model 6), or in combination with fat mass (Model 7). In contrast, the inverse association between FN BMD and low-impact activity was attenuated by approximately 25% after adjustment for fat mass either alone or in combination with lean mass, whereas there was minimal attenuation after adjustment for lean mass alone. There was little effect of additional adjustment for socioeconomic position (Model 8).

#### Adjustment for other activity bands: three-band models

We explored the suggestion from these analyses that 4.2*g* represents the best estimate for threshold value, and that impacts below this range have little positive relationship with BMD. Analyses were repeated using a three-band model, incorporating a medium band between 2.1 and 4.2*g*, in addition to high and low bands above and below this range, respectively. In minimally adjusted analyses, there was albeit weak evidence of a positive association between FN BMD and impacts in the range 2.1 to 4.2*g* (Model 1, Table 5), but this was completely attenuated after adjustment for impacts in lower and higher bands (Model 4). Equivalent results were obtained based on middle bands of 2.1 to 3.1*g* and 3.1 to 4.2*g* (results not shown). Additional adjustment for fat and lean mass attenuated the positive association with high-impact activity by approximately 35% (Model 7). There was little effect of additional adjustment for socioeconomic position (Model 8).

**Table 5:** Relationships Between Femoral Neck BMD and the Number of Counts Within Low, Medium, and High *g*-Bands

	Low (0.5–2.1*g*)			Medium (2.1–4.2*g*)			High (>4.2*g*)			
				
Model	beta	95%CI	*p*	beta	95%CI	*p*	beta	95%CI	*p*	*R*^2^
1	−0.005	−0.0668, 0.0595	0.888	0.046	0.0004, 0.0935	0.050	0.079	0.0339, 0.1218	<0.001	−
4	−0.052	−0.1352, 0.0322	0.223	−0.058	−0.1448, 0.0307	0.195	0.137	0.0646, 0.2087	<0.001	0.135
7	−0.048	−0.1239, 0.0304	0.223	−0.020	−0.0988, 0.0601	0.623	0.090	0.0159, 0.1633	0.015	0.258
8	−0.045	−0.1264, 0.0357	0.289	−0.038	−0.1229, 0.0474	0.393	0.096	0.0155, 0.1696	0.016	0.261

g-Bands are defined as low (0.5–2.1*g*), medium (2.1–4.1*g*), and high (>4.2*g*) bands. Values are for 724 boys and girls combined. Data is shown as beta (SD change in BMD per doubling of activity) with 95%CI, *p* values, and *R*^2^ for overall fit for models 2, 3, and 4 (bootstrap estimates derived from 1000 replicates). Model 1 is adjusted for age, height, and gender; model 4 = model 1 + physical activity; model 7 = model 4 + fat and lean mass; model 8 = model 7 + social position (*n* = 588). Model 1 has three separate *R*^2^ values: 0.5–2.1*g* = 0.111; 2.1–4.2*g* = 0.114; >4.2*g* = 0.126. Tests for gender interaction all *p* > 0.05.

BMD = bone mineral density; CI = confidence interval.

### Impacts associated with different running speeds

To explore the type of activity associated with our putative threshold of >4.2*g*, we reanalyzed data obtained in our previous calibration study (K Deere and colleagues, unpublished data), by comparing the number of counts within low (<2.1*g*), medium (2.1–4.2*g*), and high (>4.2*g*) bands according to running speed, in 22 adolescents performing a supervised jogging/running activity. Participants who ran at >10 km/h accrued approximately six times more high impact counts compared to those running more slowly (Supplementary Table 2).

## Discussion

We performed a population-based study of the relationship between extent of physical activity in adolescents, partitioned according to six levels of impact, and hip BMD. The number of impacts within the highest bands was positively related to BMD at all hip sites, with only minimal attenuation after adjustment for a range of potential confounding factors. Initial analyses suggested a threshold effect, since there was only evidence of a positive association between extent of activity and hip BMD for impacts >2*g*. Due to the close relationship between counts within adjacent impact bands, this estimate may be relatively imprecise; further analyses adjusted for other activity types suggested that the true threshold is considerably higher. For example, in analyses based on a three-band model in which impacts were divided into low (0.5–2.1*g*), medium (2.1–4.2*g*), or high (>4.2*g*), adjusted for impacts in other bands, a doubling in high-impact activity was associated with a 0.14 SD increase in FN BMD, whereas there was no evidence of a positive association with low or medium impacts. However, these analyses need to be interpreted with some caution due to the near colinearity of medium- and high-impact activity, which can have the effect of magnifying reciprocal changes in beta coefficients after adjusting for closely correlated variables. Therefore, although our results suggests that impacts beyond 4.2*g* exert a positive influence on hip BMD, we are unable to exclude the possibility that lesser impacts also exert an albeit weak positive effect.

Activity data is somewhat skewed, such that an individual at the 75th percentile performs approximately fivefold more high-impact activity (ie, >4.2*g*) compared to an individual at the 25th percentile, a difference which on the basis of our findings would be expected to result in an increase in FN BMD of approximately 0.3 SD. Given that a 1.0 SD decrease in FN BMD is thought to be related to approximately a 50% decrease in fracture risk,^15^ this gain is expected to translate into a reduction in fracture risk of about 15% on moving from 25th to 75th percentile for high-impact PA. In terms of which activities are most likely to produce gains in hip BMD, a previous calibration study using a similar device in premenopausal women revealed that activities such as running and jumping generate high impacts (3.9–5.3*g*), in contrast to jogging (2.5–3.8*g*).^11^ We obtained equivalent results based on our own calibration study. For example, drop jumps from a height of 38 cm were associated with impacts above 5*g*, and running at speeds above 10 km/h led to impacts above 4.2*g*. Whereas all participants in the present study recorded an average of at least one impact per day >4.2*g*, based on our calibration study, running at >10 km/h for 500 m per day would move an individual from the 25th to the 75th percentile (see Supplementary Table 2).

In light of previous evidence that FN BMD is protective against hip fracture in elderly women,^15^ to the extent that relationships with physical activity that we observed persist into later life, our findings suggest that greater exposure to high-impact activity in adolescence (eg, running and jumping) may be related to lower risk of hip fracture in later life. In contrast, lesser activities (eg, jogging) may have little benefit. This conclusion supports strategies designed to promote exercise interventions in childhood, of which positive effects on bone mineral accrual were noted at several sites including the hip, based on a review of 22 studies.^5^ Identification of a specific *g* threshold that needs to be exceeded may be helpful in standardizing these interventions, which currently vary widely. However, on the basis of our results, interventions focusing on high-impact activities such as jumping are most likely to be effective, in keeping with reports of positive effects of a school-based jumping intervention on hip BMD.^16^, ^17^

We are not aware of any previous population-based study of the relationship between physical activity as assessed by measuring impacts as described here, and hip BMD or other skeletal outcomes, in either adults or children. However, our findings are in keeping with findings from an exercise intervention study in 64 premenopausal women, in whom a relationship between number of impacts as assessed using a similar device to that used here and gain in hip BMD was only observed for impacts >3.9*g*.^18^ The suggestion that a threshold effect at the level we identified persists into adulthood is also consistent with findings from recent meta-analyses of exercise intervention studies in premenopausal^19^ and postmenopausal^20^ women, which concluded that regimes which aim to produce impact loading using activities such as jumping are most likely to be effective at improving hip BMD.

The observation that a threshold of exercise intensity exists which needs to be exceeded before benefiting the skeleton is consistent with our previous studies in ALSPAC, based on the Actigraph accelerometer.^9^ However, the upper range of acceleration that can be detected by the Actigraph is 2.5*g*,^10^ and so use of this instrument considerably underestimates any threshold value. Likewise, whereas our results are also in keeping with a recent study based on 380 healthy Spanish adolescents, in which the extent of moderate or vigorous physical activity (MVPA) as assessed by Actigraph was found to be positively related to FN BMD,^21^ the latter study provides little information about how intense activity needs to be for such an association to be observed.

High-impact activity was also found to affect hip structure as assessed by HSA, as shown by positive relationships with FNW and CSA, suggesting stimulation of periosteal growth, and with CT, suggesting inhibition of endosteal expansion. These changes were associated with an increase in predicted hip strength as reflected by CSMI, and appeared to be independent of associated changes in fat and lean mass, with the possible exception of CT. Overall, these findings are in keeping with previous studies suggesting that physical activity is positively related to periosteal expansion in childhood and adolescence, but inversely related to endosteal expansion, the changes in which presumably also underlie the relationships observed with hip BMD. For example, in previous studies based on HSA, a school-based jumping intervention was found to increase CSA and reduce endosteal diameter in 10-year-old girls.^22^ Furthermore, duration of MVPA as measured by Actigraph was found to be positively related to CSA in 468 children studied repeatedly between age 4 and 12 years.^23^ Equivalent results have also been obtained based on tibial pQCT. For example, a bone loading intervention study in young children was previously found to increase tibial circumference.^24^ In a previous cross-sectional study based on ALSPAC, the amount of vigorous activity as assessed by Actigraph was positively related to periosteal circumference at age 15 years, and inversely related to endosteal circumference, whereas moderate activity was unrelated to these parameters.^9^ Similarly, the amount of high-impact activity as assessed by a questionnaire asking about participation in sporting activity was found to be positively related to tibial cross-sectional area in young adult men.^6^

An unexpected observation was that in analyses adjusted for other activity, low-impact activity was inversely associated with FN BMD, particularly in our two-band model. One potential explanation for this finding is that whereas low-impact activity may have little direct effect on the skeleton, it may exert an indirect negative influence as a consequence of reductions in fat mass,^25^ of which the latter is known to be positively related to bone mass.^26^ Consistent with this possibility, the inverse association between lower-impact activity and FN BMD was partially attenuated by adjustment for fat mass, and the *p* value for the association was now >0.05. Alternatively, participants who performed large amounts of low-impact relative to high-impact activity might have lower lean mass, which could also explain these findings in view of the strong positive relationship between lean and bone mass. However, against this possibility, little attenuation was seen after adjustment for lean mass alone. In terms of other possible confounders, further adjustment for socioeconomic position did not appear to affect this association.

## Limitations

One of the limitations of this study was its cross-sectional design. Although we adjusted for a range of potential confounders, including markers of socioeconomic status, we are unable to exclude the influence of other confounders that were not measured. For example, individuals with greater exposure to high-impact PA as assessed in the present study may have participated in very different activities in earlier life; based on the present study design, it is difficult to distinguish the relative contributions of past and present activity to the associations with hip BMD that we observed. Another limitation is that in using DXA-based measures of BMD, it is difficult to distinguish effects of high impacts on volumetric BMD from those on cortical thickness and overall bone size. A further limitation was that by the nature of the Newtest output, adjacent activity bands were closely correlated, and the evidence favoring any one threshold over an adjacent one was relatively weak, highlighting the need for replication. A further limitation was that compliance with home accelerometer recordings in this age group was relatively poor. To ensure sufficient numbers were included, we used a threshold for accepting a valid recording of only 2 days. This may have been a particular problem in terms of obtaining representative values for rare high-impact events. However, this limitation is likely to have reduced the power and precision of the study, rather than introducing any bias. Finally, participants involved in this study were a selected group that are not necessarily representative of ALSPAC as a whole. Evidence that participants were of higher social class than other ALSPAC participants who were not included is consistent with this view. However, results such as associations between activity and body composition are unlikely to have been affected given these were unaffected by social class adjustment.

## Conclusions

We report the first population-based study of the relationship between hip BMD, or indeed any bone outcome, and physical activity as measured by partitioning the output from accelerometry into a range of impact bands. We found that >4.2*g* impacts were positively related to hip BMD, suggesting that high-impact activities like running produce BMD gains of the lower limb. In contrast, impacts below this threshold may have little benefit, suggesting that strains associated with moderately high-impact activities such as jogging have relatively little effect on BMD. Whereas these studies were performed in adolescents, further investigations are justified to determine whether equivalent thresholds apply to the remainder of the life-course.
