# Supplementary material for: Habitual Levels of High, But Not Moderate or Low, Impact Activity Are Positively Related to Hip BMD and Geometry: Results From a Population-Based Study of Adolescents
Source: J Bone Miner Res. 2012 Apr 10;27(9):1887–95. doi: 10.1002/jbmr.1631 (PMC3465797; doi:10.1002/jbmr.1631)
Supplement: Supplementary file 1 [file jbmr0027-1887-SD1.doc]

Supplementary Table 1

|  | **Participants** | | | | | | **Remainder of cohort** | | | | | | |
| --- | --- | --- | --- | --- | --- | --- | --- | --- | --- | --- | --- | --- | --- |
| **Body Composition** | **N** | **mean** | **(sd)** | | | | **N** | **mean** | | **(sd)** | | | |
| Fat mass (Kg) | 634 | 8.5 | (5.0) | | | | 6608 | 8.6 | | (5.2) | | | |
|  |  |  |  | | | |  |  | |  | | | |
| Lean mass (Kg) | 634 | 24.4 | (3.1) | | | | 6608 | 24.6 | | (3.2) | | | |
|  |  |  |  | | | |  |  | |  | | | |
| **Maternal Social class** | **N** | **(%)** | |  | | **N** | | **(%)** | | |  | | |
| I | 53 | (9.0) | |  | | 538 | | | (5.7) | | |  | |
| II | 211 | (35.9) | |  | | 2,931 | | | (31.2) | | |  | |
| III† | 250 | (42.5) | |  | | 4,024 | | | (42.8) | | |  | |
| III‡ | 27 | (4.6) | |  | | 756 | | | (8.0) | | |  | |
| IV | 40 | (6.8) | |  | | 943 | | | (10.0) | | |  | |
| V | 7 | (1.2) | |  | | 213 | | | (2.3) | | |  | |
| Total | 588 |  | | |  | 9405 | |  | | | | |  |
| † non-manual. ‡ manual | | |  | | | |  |  | |  | | | |

Comparison of body composition and maternal social class between the study population and ALSPAC as a whole. Anthropometry measures are based on attendees to the first research clinic for the whole cohort (age 9.8 years), in whom DXA-based measures of body composition were available in 7242 participants. Maternal social class is based on questionnaire data at 32 weeks gestation, which was available in 9993 participants.

**Supplementary Table 2**

| **Speed (km/h)** | | **N** | **Low (0.5 - 2.1g)** | **Medium (2.1 - 4.2g)** | **High (>4.2g)** |
| --- | --- | --- | --- | --- | --- |
| < 10 |  | 8 | 484 | 207 | 5 |
| 10 - 12 |  | 8 | 382 | 138 | 27 |
| > 12 |  | 6 | 285 | 195 | 38 |

Table showing median counts per g-band during jogging/running activity, running for 474m, stratified by speed, in 22 participants
